# Systemic Medication and Intraocular Pressure in a British Population: The EPIC-Norfolk Eye Study

**DOI:** 10.1016/j.ophtha.2014.02.009

**Published:** 2014-08

**Authors:** Anthony P. Khawaja, Michelle P.Y. Chan, David C. Broadway, David F. Garway-Heath, Robert Luben, Jennifer L.Y. Yip, Shabina Hayat, Nicholas J. Wareham, Kay-Tee Khaw, Paul J. Foster

**Affiliations:** 1 Department of Public Health and Primary Care, Institute of Public Health, University of Cambridge School of Clinical Medicine, Cambridge, UK; 2 Division of Genetics and Epidemiology, UCL Institute of Ophthalmology, London, UK; 3 Department of Ophthalmology, Norfolk & Norwich University Hospital, Norwich, UK; 4 NIHR Biomedical Research Centre, Moorfields Eye Hospital NHS Foundation Trust and UCL Institute of Ophthalmology, London, UK; 5 MRC Epidemiology Unit, Institute of Metabolic Science, Addenbrooke's Hospital, Cambridge, UK

**Keywords:** BMI, body mass index, BP, blood pressure, EPIC, European Prospective Investigation into Cancer, IOP, intraocular pressure, SPB, systolic BP, ORA, Ocular Response Analyzer

## Abstract

**Objective:**

To determine the association between systemic medication use and intraocular pressure (IOP) in a population of older British men and women.

**Design:**

Population-based, cross-sectional study.

**Participants:**

We included 7093 participants from the European Prospective Investigation into Cancer–Norfolk Eye Study. Exclusion criteria were a history of glaucoma therapy (medical, laser, or surgical), IOP asymmetry between eyes of >5 mmHg, and missing data for any covariables. The mean age of participants was 68 years (range, 48–92) and 56% were women.

**Methods:**

We measured IOP using the Ocular Response Analyzer. Three readings were taken per eye and the best signal value of the Goldmann-correlated IOP value considered. Participants were asked to bring all their medications and related documentation to the health examination, and these were recorded by the research nurse using an electronic case record form. The medication classes examined were angiotensin-converting enzyme inhibitors, angiotensin-receptor blockers, α-blockers, β-blockers, calcium channel blockers, diuretics, nitrates, statins, insulin, biguanides, sulfonylureas, aspirin, and other nonsteroidal anti-inflammatory drugs. We examined associations between medication use and IOP using multivariable linear regression models adjusted for age, sex, and body mass index. Models containing diabetic medication were further adjusted for glycosylated hemoglobin levels.

**Main Outcome Measures:**

Mean IOP of the right and left eyes.

**Results:**

Use of systemic β-blockers (−0.92 mmHg; 95% CI, −1.19, −0.65; *P*<0.001) and nitrates (−0.63 mmHg; 95% CI, −1.12, −0.14; *P* = 0.011) were independently associated with lower IOP. The observed associations between statin or aspirin use with IOP were no longer significant after adjustment for β-blocker use.

**Conclusions:**

This is the first population-based study to demonstrate and quantify clinically significant differences in IOP among participants using systemic β-blockers or nitrates. Lower IOP observed in participants using statins or aspirin was explained by concurrent systemic β-blocker use. The study findings may have implications for the management of glaucoma patients with comorbidity, and may provide insight into the pathophysiologic processes underlying IOP.

Raised intraocular pressure (IOP) is an important risk factor for the incidence^1^ and progression^2^ of glaucoma. The risk of developing open-angle glaucoma in a healthy population has been shown to increase by 16% per 1-mmHg increase in IOP.^1^ Little is known regarding the influence of systemic medication on IOP, other than for β-blockers.^3–5^ If a systemic medication does have an influence on IOP, this may give insight into the physiologic or pathologic mechanisms underlying IOP, and may aid the management of glaucoma patients with systemic comorbidity. Furthermore, for systemic medications found to have an influence on glaucoma risk, such as statins^6,7^ or calcium channel blockers,^8^ it would be of interest to know whether these medications influence IOP, or whether their effect on glaucoma risk is largely IOP independent. To date, no population-based study has systematically examined the association between common classes of systemic medication and IOP.

The aim of this study was to examine the association between the use of common systemic medications and IOP in a British population.

## Methods

### Participants

The European Prospective Investigation into Cancer (EPIC) study is a pan-European prospective cohort study designed to investigate the etiology of major chronic diseases.^9^ EPIC-Norfolk, one of the UK arms of EPIC, recruited and examined 25,639 participants aged 40 to 79 between 1993 and 1997 for a baseline examination.^10^ Recruitment was via general practices in the city of Norwich and the surrounding small towns and rural areas, and methods have been described in detail previously.^10^ Because virtually all residents in the United Kingdom are registered with a general practitioner through the National Health Service, general practice lists serve as population registers. Ophthalmic assessment formed part of the third health examination and this has been termed the EPIC-Norfolk Eye Study.^11^ In total, 8623 participants were seen for the ophthalmic examination, between 2004 and 2011. The EPIC-Norfolk Eye Study was carried out following the principles of the Declaration of Helsinki and the Research Governance Framework for Health and Social Care. The study was approved by the Norfolk Local Research Ethics Committee (05/Q0101/191) and East Norfolk & Waveney NHS Research Governance Committee (2005EC07L). All participants gave written, informed consent.

### Measurements

We measured IOP with a noncontact instrument, the Ocular Response Analyzer (ORA; Reichert, Corp, Buffalo, NY). The ORA uses a short (20-ms) pulse of air to indent the cornea and measure inward and outward applanation pressures using an electrooptical system.^12^ The average of inward (P1) and outward (P2) applanation forces has been calibrated to derive a measure equivalent to IOP measured by Goldmann applanation tonometry; this is termed Goldmann-correlated IOP.^13^ In this study, 3 ORA readings were taken per eye and the best signal value of the Goldmann-correlated IOP used (based on the best quality pressure waveform as assessed by the ORA software). Height and weight were measured with participants wearing light clothing and no shoes. Height was measured to 0.1 cm using a stadiometer, and weight was measured to the nearest 0.1 kg using digital scales (Tanita UK Ltd, Middlesex, UK). Body mass index (BMI) was calculated as weight/height^2^. Blood pressure (BP) and heart rate were measured with the participant seated resting using an objective measurement device (Accutorr Plus; Datascope Patient Monitoring, Mindray UK, Ltd, Huntington, UK) on 2 separate occasions during the health examination and the mean of the 2 measurements considered. Participants were asked to bring all their medications and related documentation to the health examination, and these were recorded by the research nurse using an electronic case record form.

### Statistical Analysis

The classes of medication to be tested were decided a priori, based on the most common medications taken in the cohort; these were angiotensin-converting enzyme inhibitors, angiotensin-receptor blockers, α-blockers, β-blockers, calcium channel blockers, diuretics, nitrates, statins, diabetic medication (insulin, biguanides, and sulfonylureas), aspirin, and nonsteroidal anti-inflammatory drugs excluding aspirin. Lists of the medications in these classes are provided in Appendix 1 (available at www.aaojournal.org).

The mean IOP of the right and left eyes of each participant was used for analyses. If data were only available for 1 eye, then the IOP of that eye was considered for the participant. Participants with an intereye IOP difference of >5 mmHg were excluded from analyses, because the asymmetry may have been caused by undetected ocular disease or may have been owing to an artifact. We further excluded participants reporting a history of glaucoma medication or a glaucoma procedure. Comparisons of IOP in participants taking medication versus those not taking medication were undertaken for each class of drug using the independent samples *t* test. To test whether any differences in IOP were independent of possible confounders, we used multivariable linear regression models with IOP as the dependent variable, and medication, age, gender, and BMI as explanatory variables. Models containing diabetic medications were further adjusted for blood glycosylated hemoglobin level. Considering the multiple statistical tests conducted and the exploratory nature of these analyses, we highlighted results significant at the 5% level after Bonferroni correction.

Given that many participants were taking >1 class of medication, we repeated regression analyses further adjusting for a particular class of drug, 1 at a time, for each drug found to be significantly associated with IOP in the original regression analyses. We also included all drugs found to be significant in individual analyses together in 1 multivariable regression model, adjusted for possible confounders.

To determine whether any association between antihypertensive medication and IOP was mediated by a change in heart rate or BP, we repeated regression analyses further adjusted for heart rate, and systolic BP (SBP) or diastolic BP.

Stata version 12.1 (StataCorp LP, College Station, TX) was used for all statistical analyses.

## Results

Of the 8623 participants attending the Eye Study, there were complete data for IOP and covariables from 7650 participants after exclusion of participants reporting a history of glaucoma medication use (n = 276) or a glaucoma procedure (n = 66). After further excluding participants with an intereye IOP asymmetry of >5 mmHg (n = 557), there were data from 7093 participants (82% of those attending the Eye Study) that were used for the main analyses. The mean age of included participants was 68 years (range, 48–92) and 56% were women. Compared with included participants, excluded participants were significantly older (*P*<0.001), had higher SBP (*P* = 0.008), and more were men (*P* = 0.002). Included and excluded participants did not have significantly different BMI (*P* = 0.74) or heart rate (*P* = 0.29).

Table 1 summarizes the number of participants taking each class of medication at the time of the health examination, and provides a comparison of the mean IOP between those taking and not taking each medication. Participants taking β-blockers (*P*<0.001), nitrates (*P*<0.001), statins (*P* = 0.002), or aspirin (*P*<0.001) had lower IOP on average than participants not taking each medication. Participants using biguanides or sulfonylureas had a higher IOP on average than participants not taking the medication, although there were no significant differences after correction for multiple testing.

**Figure 1:**
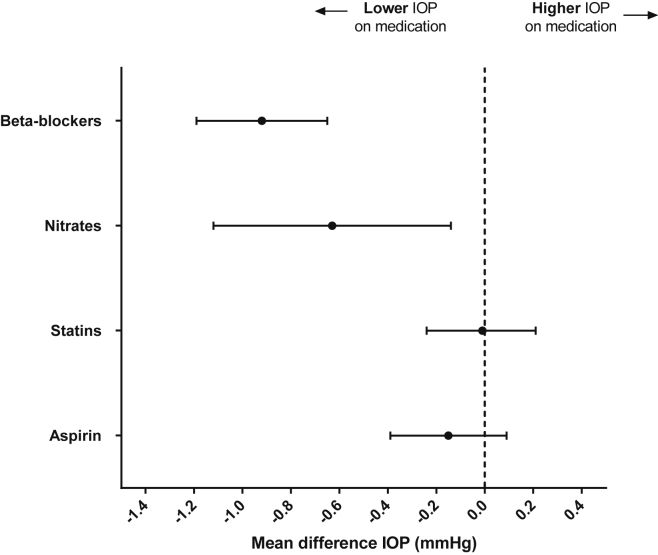
Regression coefficients with 95% confidence intervals for the associations between medication classes and intraocular pressure (IOP). These are results from 1 multivariable regression model containing all medications shown and further adjusted for age, gender, and body mass index.

After adjustment for possible confounders (age, gender, BMI, and blood glycosylated hemoglobin level), β-blocker (*P*<0.001), nitrate (*P*<0.001), statin (*P* = 0.003), and aspirin (*P*<0.001) use remained significantly associated with lower IOP (Table 2). When these 4 drugs were included in the same multivariable model, only the use of β-blockers (−0.92 mmHg; 95% CI, −1.19 to −0.65; *P*<0.001) or nitrates (−0.63 mmHg; 95% CI, −1.12 to −0.14; *P* = 0.011) remained significantly associated with IOP (Fig 1). Further analysis identified concurrent use of β-blockers as the explanation for the single medication associations observed between IOP and statins or aspirin; these associations lost significance when further adjusted for β-blocker use, but remained significant after further adjustment for nitrate use (Appendix 2; available at www.aaojournal.org).

The magnitude of IOP-lowering associated with systemic β-blocker or nitrate use was reduced after further adjustment for SBP or HR, but remained significant (Table 3). Results were similar if adjustment was for diastolic BP rather than SBP (Table 3).

## Discussion

In this population-based study of older British people, we found both systemic β-blocker use and nitrate use to be associated with a lower IOP. The association between statin or aspirin use and IOP seemed to be explained by the concurrent use of systemic β-blockers. This study is the first to report the associations of systemic β-blocker or nitrate use with IOP in a population-based sample.

After the discovery that intravenous propranolol lowered IOP in the 1960s,^3^ there have been several studies demonstrating the ocular hypotensive effect of systemic β-blockers.^4,5,14–19^ However, these studies were mostly trials in small numbers of patients with minimal follow-up periods. Oral β-blockers are commonly used in the management of cardiovascular disorders,^20^ and 12% of this study population reported use of oral β-blockers. It would be of interest to better understand the effect of longer term oral β-blocker use on IOP at a population level, and to the best of our knowledge this has not been reported previously. We found participants using oral β-blockers to have around 1 mmHg lower IOP than those not using the medication, independently of age, gender, or BMI. A difference of 1 mmHg is relatively large on a population level and, based on 5-year incidence data from the Rotterdam Study,^1^ would translate into a 14% reduced risk of incident glaucoma. Assuming the prevalence of oral β-blocker use to be 12% (as in this study) and using incident glaucoma figures from another European population^1^ and mid-2012 UK population statistics,^21^ oral β-blocker use may account for 1022 fewer people aged >55 developing definite or probable open-angle glaucoma per year in the UK. This estimation also assumes that β-blockers have no other effect on glaucoma other than via IOP. There is evidence in the literature of a protective effect of oral β-blocker use on the development of glaucoma. In a study examining data from a UK primary care database, the prevalence of oral β-blocker use in the 5 years before diagnosis was significantly lower in glaucoma patients compared with controls.^22^ There was also a trend for a reduced risk of incident glaucoma in participants using oral β-blockers from the Rotterdam Study (odds ratio, 0.60; 95% CI, 0.30–1.02; *P* = 0.06).^8^

Systemic nitrate medication is a common and established treatment for chronic stable coronary artery disease.^23^ The effect of systemic nitrate use on IOP is not well-documented, and the evidence in the literature is dated and conflicting. In the first half of the 20th century, nitrate medication was considered to be contraindicated in patients with glaucoma, in part as a result of early studies demonstrating an increase of IOP after inhalation of amyl nitrate.^24,25^ Evidence contrary to the belief that nitrates raised IOP emerged in 1964, when a study of 34 individuals demonstrated no increase in IOP and often a transient decrease in IOP after sublingual glyceryl triturate or oral pentaerythritol tetranitrate.^25^ In a later study, it was reported that oral administration of 40 mg isosorbide dinitrate twice daily resulted in a reduction of IOP lasting for 6 hours in normal individuals and in patients with open- or closed-angle glaucoma.^24^ However, in a more recent masked, randomized, crossover trial of a single oral dose of isosorbide mononitrate in 10 healthy volunteers, no significant change in IOP was observed in comparison with placebo.^26^ Our study is the first to report the association between nitrate medication use and IOP at a population level, and to demonstrate that the effect is statistically independent of changes in BP or heart rate. We found participants taking nitrate medication to have around 1 mmHg lower IOP than those not taking the medication, reducing to around a 0.7-mmHg difference after taking oral β-blocker use into account. Again, this magnitude of IOP-lowering is relatively large on a population level, translating to a 10% decreased risk of incident glaucoma, or 200 fewer people aged >55 years developing definite or probable open-angle glaucoma per year in the UK, based on the assumptions we have detailed herein. The confidence intervals were wider for the nitrate effect estimate compared with the β-blocker effect estimate, likely owing to the fewer number of participants using nitrates.

Given the known association of IOP with BP and heart rate,^27,28^ it is of interest to know how much of the IOP-lowering associated with an antihypertensive medication is mediated via a reduced BP or heart rate. We found the IOP-lowering associated with systemic β-blocker or nitrate use to reduce by around one-third after adjusting for BP and heart rate (Table 3). The residual significant associations suggest IOP-lowering mechanisms of β-blockers and nitrates that are, in part, independent of BP or heart rate.

We did not find an association between statin use and IOP that was independent of oral β-blocker use. In other words, the lower IOP we observed in participants using statins was owing to these participants being more likely to have been using oral β-blockers than participants not taking statins. Several studies have reported a protective effect of statins on the development or progression of glaucoma^6,7,29,30^; however, none of these studies specifically adjusted for systemic β-blocker use. One study adjusted for overall antihypertensive medication use^6^ (which may not sufficiently account for the effect of β-blockers) and another study did not include systemic β-blocker use in the final regression model because it did not attain significance in univariable analysis (although it is not clear whether there was a significant age-adjusted effect, which would have warranted inclusion in the multivariable model).^30^ In 2 other studies, there was no consideration of systemic β-blocker use.^7,29^ Therefore, it is possible that the reported protective association of statins for glaucoma is owing to confounding by the IOP-lowering effect of concurrent systemic β-blocker use. Alternatively, statins may exert a protective effect via IOP-independent mechanisms, such as neuroprotection of retinal ganglion cells by decreasing glutamate-mediated cytotoxicity.^31^

We did not find an association between aspirin use and IOP that was independent of oral β-blocker use. This is in keeping with the results of a randomized, crossover trial of aspirin versus placebo^32^ and also in agreement with results from the Rotterdam Study, which reported that use of anticoagulants or platelet aggregation inhibitors was not associated with incident open-angle glaucoma.^33^ The Rotterdam Study did report a nonsignificant trend towards lower IOP in participants taking aspirin (−0.21 mmHg; 95% CI, −0.44 to 0.01), although this was not adjusted for concurrent oral β-blocker use.^33^

There are several implications of the results of our study. It is important to consider systemic medication when assessing patients with glaucoma or suspected glaucoma. An individual using a systemic β-blocker or nitrate may have had higher IOP for many years before the commencement of their systemic medication. Similarly, commencement or cessation of systemic medication may have implications for the management of an established glaucoma patient, resulting in an increased or decreased requirement for IOP-lowering.

Another consideration concerns the dual prescribing of oral and topical β-blockers. The majority of the observed association of oral β-blocker use and IOP in our study was independent of SBP and heart rate, and is likely to have been via the same mechanism as topical β-blockers. Furthermore, topical β-blockers are well-absorbed systemically via the nasal mucosa and via pulmonary absorption of inhaled drug particles, avoiding first-pass metabolism in the liver and resulting in high plasma levels of the drug.^34^ It follows that the IOP-lowering effect of topical β-blockers may be blunted in patients already using oral β-blockers, while increasing the chance of systemic side effects. Data from a combination of 2 randomized trials suggest that systemic β-blocker therapy reduces the effect of topical timolol.^35^ Sublingual timolol has been shown to be almost as effective as topical timolol in a randomized, crossover study of 12 patients with ocular hypertension.^14^ Contrary evidence comes from a study of 30 patients with systemic hypertension treated by oral β-blockers, which demonstrated further reduction of IOP after additional topical instillation of timolol in all patients.^36^ Either way, it has been suggested that concurrent prescription of oral and topical β-blockers is not optimal practice.^37^

It is also important to consider the effect of systemic medication on the risk for glaucoma, rather than just the effect on IOP. Although lowering the IOP would likely reduce the risk of glaucoma, the reduction in BP that occurs with β-blockers and nitrates may reduce perfusion to the optic nerve, which may in turn increase the risk of glaucoma. Certainly, lower BP has been associated with an increased prevalence^38,39^ and incidence^40^ of open-angle glaucoma in population-based studies.

The possible IOP-lowering effect of nitrate medication has not been discussed in depth in the literature and may not be well-known in clinical practice. Understanding how nitrates might lower IOP may provide insight into the pathophysiologic processes that underlie IOP and ocular hypertension, and could potentially lead to new therapies. Metabolic transformation of nitrate moieties in nitrate medications results in nitric oxide formation.^41^ Nitric oxide is an important mediator of ocular homeostatic processes, including the regulation of aqueous flow, and potential sites for action include the trabecular meshwork and ciliary body.^42–45^ It is conceivable that nitrates administered topically may be a means of lowering IOP. In a masked, randomized trial of topical isosorbide mononitrate drops in 10 patients with glaucoma or ocular hypertension, there were no changes in IOP or aqueous flow compared with placebo.^46^ However, in a more recent study in rabbits with carbomer-induced glaucoma, topical isosorbide mononitrate did lower IOP compared with vehicle, albeit to a lesser degree than topical dorzolamide.^47^ Latanoprostene is a topical nitric oxide-donating prostaglandin F2α analog currently undergoing a phase 3 trial in patients with open-angle glaucoma or ocular hypertension (registered at www.clinicaltrials.gov; identifier NCT01895972).

The strengths of this study include the population-based design and the large sample size. Given the smaller numbers of participants taking nitrate medications, the effect may not have been apparent in a smaller sample. There are some limitations of this study. The sample, although population based, was likely to have been healthier than the general population given the nature of the study, which required travel to the research clinic. Furthermore, participants we excluded from the analyses were known to be older, with higher SBP, and more were men, compared with those included in the analyses. The effect of excluding these participants would be to reduce power to detect associations unless the direction of association in the excluded participants was opposite to the direction in included participants, which is unlikely. Although it seems reasonable to assume the associations seen would apply in glaucoma patients as well, we cannot make this assertion given that the participants were largely free of ocular disease. We measured IOP at only 1 time point, and we were therefore unable to determine whether systemic medication was associated with other attributes of IOP, such as diurnal fluctuation or peak IOP. The observational and cross-sectional design of the study limits any causal inference from our findings. For example, the observed associations might be influenced by unmeasured confounders.

In conclusion, we found systemic β-blocker and nitrate use to be associated with lower IOP in a population of older British men and women. The findings may have implications for the management of glaucoma patients with comorbidity, and may provide insight into the pathophysiological processes underlying IOP.

## Figures and Tables

**Table 1:** Comparison of Mean Intraocular Pressure (IOP) between Participants Taking and Not Taking a Medication, for Different Medication Categories (n = 7093)

Medication	Number Taking Medication	Mean IOP in Participants Not Taking Medication (mmHg)	Mean IOP in Participants Taking Medication (mmHg)	Difference in IOP (95% CI)	*P* values
ACE inhibitors	1132	15.93	15.86	−0.07 (−0.29, 0.15)	0.55
Angiotensin receptor blockers	455	15.91	15.98	0.07 (−0.26, 0.40)	0.68
α-Blockers	445	15.93	15.70	−0.23 (−0.56, 0.11)	0.18
β-Blockers	837	16.04	15.01	−1.03 (−1.28, −0.78)	**<0.001**
Calcium channel blockers	854	15.93	15.78	−0.15 (−0.40, 0.09)	0.22
Diuretics	1127	15.92	15.89	−0.03 (−0.25, 0.20)	0.82
Nitrates	224	15.95	14.87	−1.08 (−1.54, −0.62)	**<0.001**
Statins	1565	15.99	15.67	−0.31 (−0.51, −0.12)	**0.002**
Insulin	67	15.92	15.99	0.07 (−0.77, 0.91)	0.87
Biguanides	203	15.90	16.44	0.54 (0.05, 1.03)	0.029
Sulfonylureas	125	15.90	16.57	0.67 (0.05, 1.29)	0.033
Aspirin	1282	16.00	15.54	−0.46 (−0.67, −0.25)	**<0.001**
NSAIDs excluding aspirin	580	15.92	15.90	−0.02 (−0.32, 0.27)	0.88

ACE = angiotensin-converting enzyme; NSAIDs = nonsteroidal anti-inflammatory drugs. *P* < 0.0038 appear in boldface, and reflect a 5% significance level adjusted for multiple comparisons using the Bonferroni correction.

**Table 2:** Results from 13 Multivariable Linear Regression Models (One for Each Medication) with Intraocular Pressure as the Dependent Variable

	β	95% CI	*P* value
ACE inhibitors	−0.03	(−0.26, 0.19)	0.76
Angiotensin receptor blockers	0.07	(−0.27, 0.40)	0.69
α-Blockers	−0.15	(−0.49, 0.19)	0.40
β-Blockers	−1.04	(−1.30, −0.79)	**<0.001**
Calcium channel blockers	−0.13	(−0.38, 0.13)	0.32
Diuretics	−0.03	(−0.27, 0.20)	0.77
Nitrates	−1.04	(−1.51, −0.58)	**<0.001**
Statins	−0.29	(−0.50, −0.09)	**0.003**
Insulin	−0.34	(−1.29, 0.62)	0.49
Biguanides	0.13	(−0.46, 0.71)	0.67
Sulfonylureas	0.45	(−0.25, 1.16)	0.21
Aspirin	−0.42	(−0.64, −0.20)	**<0.001**
NSAIDs excluding aspirin	−0.05	(−0.35, 0.24)	0.72

ACE = angiotensin converting enzyme; NSAIDs = nonsteroidal anti-inflammatory drugs. All models were adjusted for age, gender, and body mass index. Models for diabetic medication (insulin, biguanides, and sulfonylureas) were further adjusted for blood glycosylated hemoglobin level. *P*<0.0038 appear in boldface, and reflect a 5% significance level adjusted for multiple comparisons using the Bonferroni correction.

**Table 3:** Results from 5 Multivariable Linear Regression Models with Intraocular Pressure as the Dependent Variable and Both β-Blocker Use and Nitrate Use Together as Explanatory Variables

No Further Adjustment		Further Adjusted for SBP		Further Adjusted for HR		Further adjusted for SBP and HR		Further adjusted for DBP and HR	
β (95% CI)	P value	β (95% CI)	P value	β (95% CI)	P value	β (95% CI)	P value	β (95% CI)	P value
β-Blockers									
−0.97 (−1.23, −0.71)	**<0.001**	−0.89 (−1.15, −0.64)	**<0.001**	−0.76 (−1.03, −0.49)	**<0.001**	−0.71 (−0.97, −0.44)	**<0.001**	−0.69 (−0.96, −0.43)	**<0.001**
Nitrates									
−0.69 (−1.17, −0.21)	**0.005**	−0.52 (−1.00, −0.05)	**0.030**	−0.66 (−1.14, −0.19)	**0.006**	−0.50 (−0.97, −0.03)	**0.038**	−0.51 (−0.98, −0.03)	**0.035**

DBP = diastolic blood pressure; HR = heart rate; SBP = systolic blood pressure. All models were adjusted for age, gender, and body mass index with any further adjustment indicated.
