# Supplementary material for: Systemic Medication and Intraocular Pressure in a British Population: The EPIC-Norfolk Eye Study
Source: Ophthalmology. 2014 Aug;121(8):1501–7. doi: 10.1016/j.ophtha.2014.02.009 (PMC4109027; doi:10.1016/j.ophtha.2014.02.009)
Supplement: Appendix 1 [file mmc1.pdf]

Appendix 1

The following are the lists of drug names used to identify which classes of drugs each participant was using.

ACE inhibitors: Captopril, Capozide, Capoten, Vascace, Cilazapril, Enalapril, Innovace, Innozide, Staril, Fosinopril, Lisinopril, Carace, Zestril, Zestoretic, Perindopril, Coversyl, Quinapril, Accupro, Accuretic, Ramipril, Tritace, Trandolapril, Gopten, Odrik.

Alpha-blockers: Baratol, Cardura, Doralese, Doxadura, Doxazosin, Hypovase, Hytrin, Indoramin, Prazosin, Terazosin, Tamsulosin, Alfuzosin, Bazetham, Flomax, Flomaxtra, Stronazon, Xatral, Tolazoline, Phentolamine, Dibenzyline, Phenoxybenzamine, Besavar, Vasran , Combodart.

Angiotensin receptor blockers: Amias, Aprovel, Candesartan, Cilexetil, Atacand, Co-Diovan, Coaprovel, Cozaar, Cozaar-Comp, Diovan, Eprosartan, Irbesartan, Losartan, Micardis, Olmetec Plus, Olmersartan, Olmetec, Sevikar, Telmisartan, Teveten, Valsartan.

Aspirin: Aspirin, Benoral, Benorylate.

Beta-blockers: Propranolol, Inderal, Acebutolol, Sectral, Atenolol, Tenormin, Co.Tenidone, Kalten, Tenoret, Tenoretic, Beta.Adalat, Tenif, Bisoprolol, Cardicor, Emcor, Carvedilol, Eucardic, Celiprolol, Celectol, Esmolol, Brevibloc, Labetalol, Trandate, Metoprolol, Betaloc, Lopresor, Nadolol, Corgard, Nebivolol, Nebilet, Oxprenolol, Trasicor, Trasidrex, Pindolol, Visken, Viskaldix, Sotalol, Beta.Cardone, Sotacor, Timolol, Betim, Moducren, Prestim.

Calcium channel blockers: Adalat, Adipine, Adizem, Amlodipine, Amlostin, Angitil, Calcicard, Cardene, Cardioplen, Coracten, Cordilox, Dilcardia, Diltiazem, Dilzem, Exforge, Felodipine, Felogen, Felotens, Fortipine, Securon, Hypolar, Isradipine, Istin, Keloc, Lacidipine, Lercanidipine, Motens, Neofel, Nicardipine, Nifedipine, Nifedipress, Nimodopine, Nimotop, Optil, Parmid, Prescal, Securon, Sevikar, Slozem, Tensipine, Tildiem, Univer, Valni, Vascalpha, Verapamil, Verapress, Vertab, Viazem, Zanidip, Zemtard, Zolvera.

Insulin: Insulin, Hypurin, Actrapid, Velosulin, Humulin, Pur-In, Insuman, NovoRapid, Humalog, Lantus, Lentard, Monotard, Semitard, Ultratard, Insulatard, Protaphane, Rapitard, NovoMix, Mixtard, Initard, Actraphane, PenMix,

Biguanides: Metformin, Glucophage, Avandamet

Sulfonylureas: Chlorpropamide, Diabinese, Glibenclamide, Daonil, Euglucon, Gliclazide, Diamicron, Glimepiride, Amaryl, Glipizide, Glibenese, Minodiab, Gliquidone, Glurenorm, Tolazamide, Tolanase, Tolbutamide, Rastinon.

Diuretics: Aldactone, Amiloride, Baycaron, Bendrofluazide, Bendroflumethiazide, Betinex, Bumetanide, Bumex, Burinex, Centyl K, Chlorothalidone, Chlorothiazide, Chlortalidone, Chlorthalidone, Co-Amilofruse, Co-Amilozide, Co-Flumactone, Cyclopenthiazide, Demadex, Diumide-K , Ontinus, Diurexan, Dryptal, Dyazide, Dytac, Dytide, Edecrin, Ethacrynic Acid, Froop, Frusemide, Frusene, Frusol, Furosemide, Hydrenox, Hydrochlorothiazide, Hydroflumethiazide, Hydrosaluric,

Hygroton, Indapamide, Kalspare, Lasilactone, Lasikal, Lasix, Mefruside, Metenix, Metenix 5, Metolazone, Natrilix, Natrillix, Navidrex, Navispare, Neo-Naclex-K, Nephril, Polythiazide, Potassium Canrenoate, Rusyde, Saluric, Sodium Edecrin, Spiroctan, Spiroctan-M, Spironolactone, Torasemide, Torem, Torsemide, Triamterene, Xipamide, Co-Triamterzide, Mannitol, Natrilix SR.

Nitrates: Angeze, Angitak, Chemydur, Coro-Nitro, Deponit, Elantan, Glyneryl Trinitrate, Glytrin, Gtn, Imdur, Isib, Ismo, Isodur, Isoket, Isosorbide Dinitrate, Isosorbide Mononitrate, Isotard, Minitran, Modisal, Monomax, Monomil, Monosorb, Nitromin, Nitro-Dur, Nitrolingual, Isoket Retard, Percutol, Suscard, Transiderm-Nitro, Zemon.

NSAIDS not aspirin: Aceclofenac, Acemetacin, Alrheumat, Apsifen, Arthrofen, Arthrotec, Azapropazone, Brufen, Butacote, Clinoril, Cuprofen, Diclofenac, Diclofenac Sodium, Diclomax, Diflunisal, Disalcid, Dolobid, Ebufac, Emflex, Etodolac, Feldene, Femafen, Fenbid, Fenbufen, Fenoprofen, Fenopron, Flexin, Flurbiprofen, Froben, Ibrufhalal, Ibular, Ibuprofen, Indocid, Indolar, Indomax, Indomethacin, Indometacin, Indomod, Inoven, Junifen, Ketoprofen, Ketorolac, Lederfen, Librofem, Lidifen, Lodine, Mefenamic.acid, Meloxicam, Migrafen, Mobic, Mobiflex, Motifene, Motrin, Nabumetone, Napratec, Naprosyn, Naproxen, Novaprin, Nurofen, Nycopren, Orudis, Oruvail, Phenylbutazone, Piroxicam, Ponstan, Preservex, Proflex, Relcofen, Relifex, Rheumacin, Rheumox, Rimafen, Seclodin, Slo.indo, Sulindac, Surgam, Synflex, Tenoxicam, Tiaprofenic, Tolectin, Tolfenamic, Tolmetin, Trilisate, Voltarol.

Statins: Atorvastatin, Cerivastatin, Crestor, Fluvastatin, Inegy, Lescol, Lipitor, Lipobay, Lipostat, Pravastatin, Rosuvastatin, Simvastatin, Statin, Zocor.
