# Supplementary material for: Systemic Medication and Intraocular Pressure in a British Population: The EPIC-Norfolk Eye Study
Source: Ophthalmology. 2014 Aug;121(8):1501–7. doi: 10.1016/j.ophtha.2014.02.009 (PMC4109027; doi:10.1016/j.ophtha.2014.02.009)
Supplement: Appendix 2 [file mmc2.pdf]

Appendix 2: Table showing results from multivariable linear regression models with intraocular pressure as the dependent variable.

	No further adjustment			Adjusted for nitrate use			Adjusted for beta-blocker use		
	β	95% CI	p-value	β	95% CI	p-value	β	95% CI	p-value
**Nitrates**	-1.04	(-1.51, -0.58)	<0.001	-	-	-	-0.69	(-1.17, -0.21)	0.005
**Beta-blockers**	-1.04	(-1.30, -0.79)	<0.001	-0.97	(-1.23, -0.71)	<0.001	-	-	-
**Statins**	-0.29	(-0.50, -0.09)	0.003	-0.21	(-0.42, -0.00)	0.045	-0.11	(-0.31, 0.10)	0.31
**Aspirin**	-0.42	(-0.64, -0.20)	<0.001	-0.33	(-0.55, -0.11)	0.004	-0.21	(-0.43, 0.02)	0.07

	Adjusted for statin use			Adjusted for aspirin use		
	β	95% CI	p-value	β	95% CI	p-value
**Nitrates**	-0.94	(-1.42, -0.47)	<0.001	-0.89	(-1.37, -0.41)	<0.001
**Beta-blockers**	-1.01	(-1.27, -0.75)	<0.001	-0.98	(-1.25, -0.72)	<0.001
**Statins**	-	-	-	-0.17	(-0.39, 0.05)	0.13
**Aspirin**	-0.35	(-0.59, -0.11)	0.004	-	-	-

All regression models were adjusted for age, sex and body mass index, and further adjusted for a particular medication as shown. Each row within each section represents a different regression model.

CI – confidence interval.
